# Association between Very Low-Density Lipoprotein Cholesterol (VLDL-C) and Carotid Intima-Media Thickness in Postmenopausal Women Without Overt Cardiovascular Disease and on LDL-C Target Levels

**DOI:** 10.3390/jcm9051422

**Published:** 2020-05-11

**Authors:** Marco Gentile, Arcangelo Iannuzzi, Francesco Giallauria, Antonello D’Andrea, Elio Venturini, Mario Pacileo, Giuseppe Covetti, Camilla Panico, Amalia Mattiello, Giuseppe Vitale, Filippo Maria Sarullo, Paolo Rubba, Carlo Vigorito, Salvatore Panico, Gabriella Iannuzzo

**Affiliations:** 1 Department of Clinical Medicine and Surgery, “Federico II” University, 80131 Naples, Italy; camillapanico@hotmail.it (C.P.); amattiel@unina.it (A.M.); rubba@unina.it (P.R.); spanico@unina.it (S.P.); gabriella.iannuzzo@unina.it (G.I.); 2 Department of Medicine and Medical Specialties, “Antonio Cardarelli” Hospital, 80131 Naples, Italy; lelliann@alice.it (A.I.); giucov@alice.it (G.C.); 3 “Federico II” University of Naples, Department of Translational Medical Sciences, 80131 Naples, Italy; francesco.giallauria@unina.it (F.G.); vigorito@unina.it (C.V.); 4 Cardiology and Intensive Care Units, “Umberto I” Hospital, Viale San Francesco, 84014 Nocera Inferiore (Salerno), Italy; antonellodandrea@libero.it (A.D.); pacmario@yahoo.it (M.P.); 5 Cardiac Rehabilitation Unit, Azienda USL Toscana Nord-Ovest, Cecina Civil Hospital, 57023 Cecina (LI), Italy; vent.elio@tin.it; 6 Cardiovascular Rehabilitation Unit, “Buccheri La Ferla Fatebenefratelli” Hospital, 90123 Palermo, Italy; giuseppevit@hotmail.com (G.V.); sarullo.filippo@fbfpa.it (F.M.S.)

**Keywords:** VLDL, atherosclerosis, carotid intima-media thickness, women, menopause

## Abstract

Background: atherosclerotic process inexorably advances in patients reaching low-density lipoprotein cholesterol (LDL-C) targets. An attractive hypothesis is that lipoprotein particles (very low-density lipoprotein (VLDL), intermediate-density lipoprotein (IDL)), could contribute to residual risk. The present study aims to investigate the relationship between carotid intima-media thickness (IMT) and different lipoprotein subfractions in a cohort of healthy postmenopausal women. Methods: 75 postmenopausal women, at LDL-C target levels without overt cardiovascular disease, underwent biochemical analyses (including subfraction assay of plasma lipoproteins) and carotid ultrasound examination. Results: a statistically significant correlation between VLDL and carotid IMT (*p* < 0.001) was found. No significant correlation was found between carotid IMT and LDL-C (*p* = 0.179), IDL-C (*p* = 0.815), high-density lipoprotein (HDL) (*p* = 0.855), and LDL score (*p* = 0.240). Moreover, IMT is significantly correlated to LDL particle diameter (*p* = 0.044). After adjusting for age, systolic blood pressure, body mass index, smoking habits, glucose plasma concentration, and Lipoprotein(a) (Lpa) levels, multivariate analysis showed that women in the third tertile of VLDL-C, compared with those in the first tertile, were significantly associated to the highest IMT (*p* = 0.04). Conclusions: in this cohort of postmenopausal women, VLDL-C was significantly associated to carotid IMT, independent of main cardiovascular risk factors. These findings pave the way for targeting circulating concentrations of VLDL-C to reduce cardiovascular events in patients with target LDL-C levels.

## 1. Introduction

The role of statins and other lipid-lowering drugs in lowering low-density lipoprotein cholesterol (LDL-C) and reducing cardiovascular disease (CVD) has been well established in trials of both primary and secondary prevention [1,2].

Despite the significant reductions in LDL-C and successive CVD reduction, residual atherosclerotic cardiovascular disease (ASCVD) persists, especially among high-risk subjects with type 2 diabetes mellitus (T2DM), metabolic syndrome, and obesity, where atherogenic dyslipidemia is associated, in particular, with elevated levels of triglycerides (TG), low levels of high-density lipoprotein cholesterol (HDL-C), and increased remnant lipoproteins. In populations with high prevalence of obesity and diabetes, LDL-C is a less predictive marker. Previous studies suggest that elevated levels of triglyceride rich lipoproteins and their remnants are associated with an increased risk of CVD [3,4]. In addition, clinical studies indicate that a focus solely on the assessment and management of LDL-C is not an optimal strategy for all patients; emerging evidence has established that very low-density Lipoprotein (VLDL), their remnants, and Lipoprotein(a) (Lpa), likewise, are causally related to CVD [5,6]. Therefore, an attractive hypothesis is that other lipoprotein particles (VLDL, intermediate-density lipoprotein (IDL)), and their remnants, Lpa, could contribute to the residual risk in subjects at LDL-C goal.

In a cohort of 228 menopausal women, after adjusting for main cardiovascular risk factors, a significant association between higher concentrations of IDL-C and cholesterol contained in triglyceride-rich lipoproteins (TG, VLDL-C, and IDL-C) with carotid plaques was reported [7]. Common carotid artery intima-media thickness (IMT) is a well-established measure of subclinical atherosclerosis and is widely used as a surrogate marker for CVD [8,9].

The present study aims at evaluating the relationship between lipid profile biomarkers (VLDL-C, LDL-C, IDL-C, and HDL) and other cardiovascular risk factors with subclinical atherosclerosis in a cohort of postmenopausal women, without overt CVD and at target LDL-C levels.

## 2. Methods

**Figure 1:**
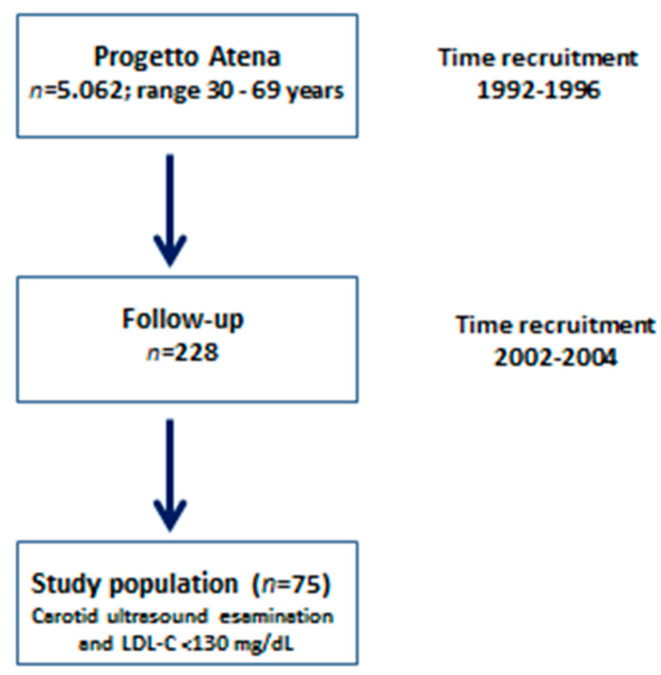
Flow diagram of selected participants.

The main purpose of the “Progetto Atena” study [10] was to investigate the causes of chronic diseases that have a major impact on the female population. The study population (*n* = 5.062; range 30–69 years) gave information on dietary habits, reproductive history, and familiarity for chronic disease, smoking habits, physical activity, and socio-demographic data. Blood pressure, anthropometry, and electrocardiogram were also taken. All of the participants provided biological samples of blood and urine. These samples were processed in order to explore the main areas under study (nutritional markers, metabolism, endocrinology, genetics, thrombogenesis, and atherosclerosis). At the 10-year follow-up, 228 women underwent follow-up clinical examinations, biochemical analyses that included Lipoprint^®^ assay (analysis of the lipoprotein subfractions), and ultrasound carotid examinations. At the time of the follow-up visits, all women were postmenopausal. In 78 out the 228 women (34.2%), LDL-C was <130 mg/dl; whereas biochemical lipoprotein subfraction analyses and carotid IMT measurements where available in 75/228 (32.8%); therefore, only 75 out of the 228 postmenopausal women were considered for the analysis and included in this cross-sectional study (Figure 1). Progetto Atena was approved by the Ethical Committee of School of Medicine of University “Federico II” in 1992. The approval included the permission, as reported in the individual consensus given by each participant, to analyze personal data for future research unless genetic data are used. 

### 2.1. Clinical and Biochemical Assessment

Body mass index (BMI), was calculated as weight (kilograms) divided by height (squared meters). Waist circumference was measured midway between the bottom of the rib cage and the top of the iliac crest. Sitting brachial blood pressure was measured two times using a random-zero sphygmomanometer; the mean value of two measurements was reported. A standardized questionnaire was used to acquire information on smoking habits [10].

Blood specimens were collected after a 12 to 14 h fast, from 8:00 to 9:30 a.m. Fasting glucose, total cholesterol (TC), triglyceride, and high-density lipoprotein (HDL) were measured using automated methods [11]. The Friedewald equation was used to calculate LDL-C from total cholesterol, HDL-C, and

TG: LDL-C = TC − HDL-C − (TG/5).(1)

Fasting ultrasensitive insulin levels were dosed by enzyme immunoassay (Mercodia, Sweden) [8]. The error of the method was < 10%. Apolipoprotein B and high-sensitivity C-reactive protein (CRP) were measured by using an automated method (Cobas-Mira, Roche, Italy) [8]. The computed error was <5%. The homeostatic assessment model index was calculated as fasting serum insulin (KU/mL) × fasting serum glucose (mM)/22.5, as described by Matthews et al. [12]. The American Heart Association Scientific Statement criteria were used to classify women as having the metabolic syndrome (MS) diagnosis [13].

### 2.2. Lipoprotein Determination

Separation of VLDL, IDL, LDL, and HDL particles on the basis of size was performed on frozen serum samples (−80°C) by using polyacrylamide 3% gel electrophoresis Lipoprint^®^ System (Quantimetrix Inc., Redondo Beach, California) [8]. 

LDL mean particle diameter and LDL score, as a proportion of small dense LDL particles (subfractions 3–7) to the whole LDL area (subfractions 1–7) were calculated [8].

### 2.3. High-Resolution Carotid Ultrasound

An expert sonographer performed high-resolution B-mode carotid ultrasound examination by using Esaote AU4 (Biosound Esaote, Inc., Indianapolis, IN, USA). The sonographer was blinded to participant biomarker data. Individuals were examined in the supine position and the head was gently tilted away from the side being scanned. The IMT was measured at the right and left common carotid artery, 1 cm proximal to the bifurcation on the anterior and posterior wall of the artery. The sonographer, following a standardized protocol, carefully and slowly turned the probe at different scanning angles (anterior, lateral, and posterior) to recognize the maximum thickness of the IMT for each wall [9]. The images were recorded for further analyses. The IMT mean–max was defined as the mean of the maximum thicknesses measured between anterior and posterior CC walls on each side. Right and left values were then averaged. In this study, we considered IMT mean–max value >1.14 mm (IMT mean–max 75th percentile) as CC thickened IMT. It was possible to measure IMT in all study participants. Quality control data in previous studies that used this protocol indicated a coefficient of reproducibility for IMT of 0.85, taking into account the variability of the medical equipment, sonographer, and reader [9].

## 3. Statistical Analysis

Statistical analyses were performed using SPSS version 19.0 (SPSS, Inc., Chicago, IL, USA). Continuous variables were described as mean and standard deviation. Categorical variables were expressed as proportion or percentage. Pearson’s correlation coefficient was used to investigate the correlation between IMT and lipid profile biomarkers. Univariate and multivariate logistic regression analysis were used to test the correlation between the IMT > 1.14 mm (75th percentile of studied population) (dependent dichotomous variable), and tertiles of lipoprotein subfractions before and after adjusting for age, systolic blood pressure, body mass index, smoking habits, glucose plasma concentration, and Lpa levels. Odds ratio (OR) for the presence of common carotid thickened wall were determined using unconditional logistic regression and 95% confidence intervals (CI) of the odds ratio were computed.

## 4. Results

The physical and biochemical characteristics of the study cohort were reported in Table 1. All included patients were on LDL-C target levels. The prevalence of overweight/obesity, following the World Health Organization classification, was 63% (33% overweight, 30% obese). About one third (*n* = 24) of women cohort had metabolic syndrome. Only 13 women (17.3%) were treated with lipid-lowering drugs (statin, nicotinic acid, resin, probucol, fibrates or others); 31/75 participants (41.3%) were treated with antihypertensive drugs.

**Figure 2:**
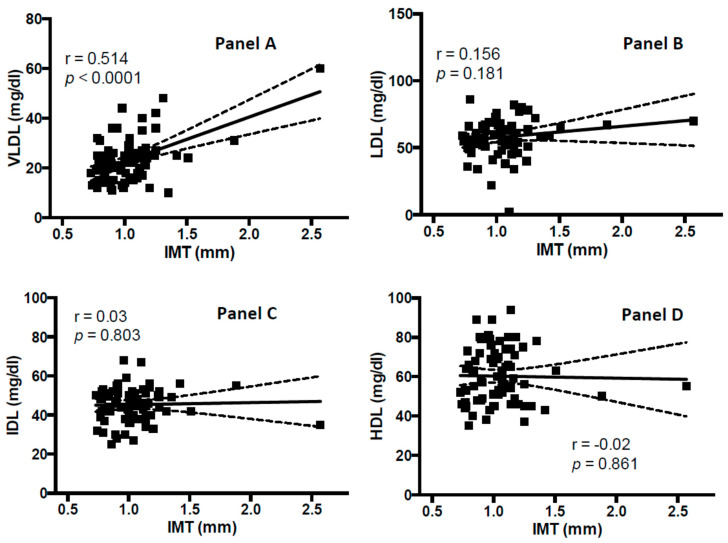
Association between carotid intima-media thickness (IMT) (mm) and lipid biomarkers: very low-density lipoprotein cholesterol (VLDL-C) (panel **A**), low-density lipoprotein cholesterol (LDL-C) (panel **B**), intermediate-density lipoprotein cholesterol (IDL-C) (panel **C**), high-density lipoprotein (HDL) (panel **D**).

**Figure 3:**
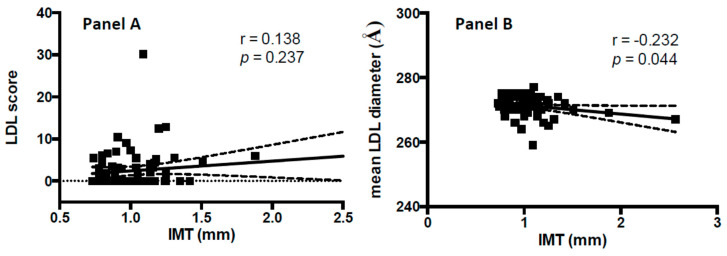
Association between carotid IMT (mm) and LDL score (panel **A**) and LDL mean particle diameter (panel **B**).

In Figure 2 the associations between IMT with VLDL-C, LDL-C, IDL-C, HDL, LDL score and mean LDL particle diameter measured with Lipoprint^®^ assay are depicted. A statistically significant correlation between VLDL-C and carotid IMT (r = 0.51, *p* < 0.001) was found (Figure 2, panel A). After adjusting for age, systolic blood pressure, body mass index, smoking habits, glucose plasma concentration, and Lpa levels, logistic regression analysis showed that VLDL-C and carotid IMT were significantly associated (B = 0.11, *p* = 0.002). No significant correlation was found between carotid IMT and LDL-C (r = 0.15, *p* = 0.179) (Figure 2, panel B), IDL-C (r = 0.02, *p* = 0.815) (Figure 2, panel C), and HDL (r = 0.01, *p* = 0.855) (Figure 2, panel D) and LDL score (r = 0.13, *p* = 0.240) (Figure 2, panel A). In addition, carotid IMT is significantly associated to LDL mean particle diameter (r = −0.232, *p* = 0.044) (Figure 3, panel B); whereas no significant association between Lpa and carotid IMT was observed (r = 0.10, *p* = 0.404). Univariate analyses showed a significant correlation between the highest IMT percentile (>75th) and the highest tertile of VLDL-C (B = 1.73, *p* = 0.02); whereas no significant association was found between the highest IMT percentile (>75th) and tertiles of other lipoprotein subfractions (Table 2). After adjusting for age, systolic blood pressure, body mass index, smoking habits, glucose plasma concentration and Lpa levels, multivariate logistic analysis showed that women in the third tertile of VLDL-C, compared with those in the first tertile were significantly associated to highest IMT (>75th percentile) (OR = 15.49, *p* = 0.04).

## 5. Discussion

The present study demonstrated an independent association between VLDL-C and carotid IMT in a cohort of postmenopausal women without overt cardiovascular disease, and with on-target LDL-C levels. LDL-C levels are declining with increasing use of lipid-lowering drugs and better adherence to healthy lifestyle [14]. However, cardiovascular disease events occur despite low levels of LDL and/or statin therapy in some individuals, a phenomenon referred to as residual risk; triglyceride-rich lipoproteins (especially VLDL) and their cholesterol content could partially explain this residual risk.

These lipoproteins are novel biomarkers driving residual cardiovascular risk in our contemporary era of elevated incidence rates of obesity, diabetes, and metabolic syndrome [15]. In particular, VLDL remnants (as well as LDL particles) have been shown to migrate across the endothelium where they are entrapped by macrophages, forming foam cells, promoting low-grade inflammation and facilitating atheromatous plaque growth [16]. There is a need to better define the relationship between plasma lipoprotein particles that carry triglycerides and cholesterol (especially VLDLs, IDLs, and their remnants), Lpa and the relative atherogenicity of those lipoproteins versus LDL. Therefore, it is important to assess the independent contribution of lipid fractions, including VLDL and Lpa, because therapeutic strategies may differ by using novel specific lipid-lowering drugs [17].

The Jupiter trial showed that atherogenic lipoprotein particle concentrations, i.e., VLDL lipoproteins, particularly the smallest remnant subclass, were associated with cardiovascular disease risk when LDL was low [18]. In this experimental study, authors underlined that chemically measured triglycerides might not sufficiently capture risk related to VLDL pathways [18].

Recently, in a large REDUCED-IT trial (*n* = 8179), authors demonstrated that the administration of icosapent ethyl, 4 g daily in patients at elevated risk for cardiovascular disease who have increased baseline TG, but well-controlled LDL-C levels, have highly significant clinical benefit and reduced incident events, including cardiovascular death over and above statin therapy [19].

In a recent study, Abi-Ayad et al. [20] evaluated the association between lipoproteins (HDL, LDL, VLDL) and apolipoproteins (ApoA1, ApoB100) with carotid plaque in patients with metabolic syndrome diagnosis, and free from cardiovascular disease (CVD). Interestingly, these authors found that carotid plaques were associated with lower levels of HDL-C, ApoA1, and high levels of VLDL-TG.

In statin-treated patients with atherosclerotic cardiovascular disease, remnant cholesterol was associated with coronary atheroma progression independently of lipid parameters, C-reactive protein, or other clinical risk factors. Higher remnant cholesterol levels, in addition, were correlated with higher major adverse cardiovascular events [21].

In a recent study [7], we demonstrated, in a group of 228 women (LDL range 72 mg–241 mg/dL), that VLDL Cholesterol and triglyceride-rich lipoproteins were independently associated with the presence of carotid plaques after correction for main cardiovascular risk factors.

In the present study, a positive and independent association between VLDL-C and IMT after controlling for major CV risk factors was found. Moreover, the highest tertile of VLDL-C was associated with increased thickness of carotid wall. The increase of carotid wall thickness is at the basis of arterial stiffening and loss of elasticity [22,23]. Arterial stiffening is one of the hallmarks of vascular aging, and is an important risk factor for cardiovascular morbidity and mortality [24,25,26,27,28]. Recent evidences suggest that arterial stiffness accelerates within 1 year of the final menstrual period [29]; therefore, subclinical atherosclerosis is progressive and aggressive in postmenopausal women; thus, increasing their cardiovascular risk. Unfortunately, data on diet habits and/or physical activity levels were not available. Healthy lifestyle during the menopausal transition is associated with less subclinical atherosclerosis, highlighting the key role, during midlife, for cardiovascular prevention in women [30].

Since subclinical atherosclerosis interfere with future clinical CVD and physical/cognitive functioning, the potential effect of modifiable health behaviors on subclinical atherosclerosis in midlife and early postmenopausal women warrants further investigation, as it may be a critical window of opportunity for prevention [31,32,33].

Interestingly, lifestyle education program targeting diet and physical activity might play a key role in slowing menopause related progression of atherosclerosis [34]. Therefore, prevention strategies aimed at counteracting residual risk and subclinical atherosclerosis should be engaged [1].

With the development of new lipid-lowering agents with highly specific targets, the simple assessment of total cholesterol or lipoproteins by measuring non-HDL-cholesterol or apo B levels could not be sufficient [17]. In particular, in individuals with LDL-C < 130 mg/dL, accurate measurement of the relative contribution of each atherogenic cholesterol fraction to cardiovascular risk may be essential to personalize lipid-lowering treatment strategies.

Several study limitations should be acknowledged. First, the relatively small sample size population, exclusively women. The cross-sectional nature of this study does not allow us to ascertain the causal relationship of the association between VLDL-C and carotid IMT. Additional studies are needed to understand prospectively this relationship. Finally, whether a correct lifestyle (diet habits plus physical activity) might interfere with this relationship should be further investigated.

In conclusion, the present study showed a significant association between VLDL-C and subclinical atherosclerosis as expressed by thicker IMT. Future studies targeting circulating concentrations of VLDL-C to reduce coronary heart disease events in patients with normal or low concentrations of LDL-C are eagerly awaited.

## Figures and Tables

**Table 1:** Clinical and biochemical characteristics of the study population.

	*n* = 75
Age (years)	61.2 ± 9.3
Total cholesterol (mg/dL)	187.4 ± 20.9
Triglycerides (mg/dL)	96.9 ± 41.1
High-density lipoprotein cholesterol (mg/dL)	59.4 ± 14.8
Low-density lipoprotein cholesterol (mg/dL)	108.6 ± 15.3
Fasting glucose (mg/dL)	102.8 ± 20.0
Apolipoprotein B (g/L)	0.9 ± 0.1
Lipoprotein a (mg/dL)	18.6 ± 23.6
Low-density lipoprotein (LDL) score (mg/dL)	2.4 ± 4.4
Mean LDL diameter (Å)	271.2 ± 3.0
High sensitive C-reactive protein (CRP) (mg/L)	3.4 ± 6.0
Creatinine (mg/dL)	0.8 ± 0.1
Body mass index (kg/m^2^)	27.9 ± 5.4
Waist circumference (cm)	89.1± 11.7
Homeostatic assessment model index (HOMA)	1.8 ± 1.3
Systolic blood pressure (mm Hg)	141.4 ± 24.3
Diastolic blood pressure (mm Hg)	80.4 ± 9.8
Intima-media thickness (mm)	1.0 ± 0.2
Active smokers (yes/not)	29.5%

Values are expressed as mean ± SD. International System conversion factors: to convert triglycerides to millimoles per liter, multiply by 0.0113; to convert high-density lipoprotein cholesterol to millimoles per liter, multiply by 0.02586; to convert glucose to millimoles per liter, multiply by 0.05551; to convert total and low-density lipoprotein cholesterol to millimoles per liter, multiply by 0.02586.

**Table 2:** Association between tertiles of lipoprotein subfractions and carotid IMT.

	OR	95%CI	*p* Value	OR	95%CI	*p* Value
	tertile II vs. tertile I (reference)			tertile III vs. tertile I (reference)		
HDL-C	0.54	0.14–2.16	0.38	0.52	0.13–2.04	0.35
LDL-C	1.27	0.25–6.40	0.77	2.81	0.65–12.18	0.17
IDL-C	2.33	0.51–10.69	0.27	2.00	0.44–9.07	0.37
VLDL-C	2.56	0.58–11.29	0.21	5.64	1.30–24.31	0.02

Dependent variable: carotid intima-media thickness (IMT). Abbreviations: HDL-C, high-density lipoprotein cholesterol; LDL-C, low-density lipoprotein cholesterol; IDL-C, intermediate-density lipoprotein cholesterol; VLDL-C, very low-density lipoprotein cholesterol; OR, Odds ratio; CI, confidence interval.
